# Infestation by pyrethroids resistant bed bugs in the suburb of Paris, France

**DOI:** 10.1051/parasite/2012194381

**Published:** 2012-11-15

**Authors:** R. Durand, A. Cannet, Z. Berdjane, C. Bruel, D. Haouchine, P. Delaunay, A. Izri

**Affiliations:** 1 Laboratoire de Parasitologie-Mycologie, Hôpital Avicenne, Assistance Publique-Hôpitaux de Paris & UFR Léonard de Vinci, Université Paris 13 Bobigny France; 2 Agence Régionale de la Santé Île-de-France Paris France; 3 Service de Parasitologie-Mycologie, Hôpital de l’Archet, Centre Hospitalier Universitaire de Nice & Inserm U895/Université de Nice- Sophia Antipolis France

**Keywords:** bed bugs, *Cimex lectularius*, pyrethroids, insecticide, resistance, punaises de lit, *Cimex lectularius*, pyréthrinoïdes, insecticide, résistance

## Abstract

Bed bugs are hematophagous insects responsible for a re-emerging and challenging indoor pest in many countries. Bed bugs infestations may have health consequences including nuisance biting, cutaneous and systemic reactions. This resurgence can probably be attributed to factors such as increased international travel and development of resistance against insecticides. Resistance against pyrethroids has been reported several times from the USA and rarely in Europe. In France, very few data on bed bugs are available. The present study aimed to assess the infestation by bed bugs of a complex of two high-rise apartment buildings in the suburb of Paris and to evaluate their susceptibility to pyrethroid insecticides. We inspected for bed bugs 192 out of 198 apartments units (97%) and interviewed their residents. 76 (39.6%) apartments were infested. Among the 97 residents living in infested apartments, 53 (54.6%) reported bed bug bites. A total of 564 bed bugs were collected in the infested units. Bioassays showed that 54 out of 143 bed bugs were resistant to pyrethroids (37.8%; 95% confidence interval: 29.9-45.7%). DNA sequencing showed that all bed bugs tested (n = 124) had homozygous L925I *kdr*-like gene mutation. The level of pyrethroid resistance found indicates that this phenomenon was already established in the site and prompts the need to reevaluate the wide use of pyrethroids to control bed bugs.

## Abstract (fr)

Les punaises de lit sont des insectes hématophages cosmopolites, responsables d’infestations ré-émergentes dans de nombreux pays. L’infestation par les punaises de lit peut avoir des conséquences sanitaires comprenant les nuisances dues aux piqûres, ainsi que des réactions locales ou générales. Cette résurgence peut probablement être attribuée à différents facteurs, tels que l’augmentation des voyages internationaux et le développement de la résistance aux insecticides. La résistances aux pyréthrinoïdes a été rapportée plusieurs fois aux États-Unis, mais rarement en Europe. En France, très peu de données sur les punaises de lit sont disponibles. L’objectif de cette étude était d’évaluer l’infestation par les punaises de lit de deux immeubles voisins, situés dans la banlieue de Paris, et de déterminer la sensibilité aux pyréthrinoïdes de ces punaises. Nous avons inspecté 192 appartements sur les 198 (97%) composant les deux immeubles et eu un entretien avec leurs résidents. 76 (39.6%) appartements étaient infestés. Parmi les 97 résidents vivant dans des appartements infestés, 53 (54,6%) ont rapporté des piqûres de punaises. Au total, 564 punaises de lit ont été collectées dans les appartements infestés. Les tests *ex vivo* ont montré une résistance aux pyréthrinoïdes pour 54 punaises sur 143 testées (37,8%; intervalle de confiance 95% : 29,9-45,7%). Le séquençage de l’ADN a montré que toutes les punaises testées (n = 124) étaient homozygotes pour la mutation L925I du gène *kdr*-like. Les niveaux de résistance aux pyréthrinoïdes observés indiquent que ce phénomène est bien installé dans le site et incitent à réévaluer l’usage intensif des pyréthrinoïdes pour contrôler l’infestation par les punaises de lit.

## Introduction

Bed bugs (*Cimex lectularius* L.) are cosmopolite hematophagous insects responsible for a challenging and re-emerging indoor pest in many countries around the world (Hwang *et al.*, 2005; Gangloff-Kaufmann *et al.*, 2006; Doggett & Russell, 2009; Levy Bencheton *et al.*, 2010; Tawatsin *et al.*, 2011). The present resurgence can probably be attributed to factors such as increased international travel and development of resistance against pesticides (Reinhardt & Siva-Jothy, 2007; Mouchtouri *et al.*, 2008; Dogget & Russell, 2009). Bed bugs infestations may have health consequences including nuisance biting, which can provoke discomfort and anxiety, and cutaneous and systemic reactions (Delaunay *et al.*, 2009; 2011; Goddard & de Shazo, 2009). Although bed bugs can harbor over 45 pathogens, there is no scientific evidence that they are involved in disease transmission (Delaunay *et al.*, 2011). Recently, bed bugs were reported carrying drug-resistant bacteria (Lowe & Romney, 2011).

Reactions to insect bites, inconvenience, and psychological distress have been the basis for lawsuits against landlords and lodging industry. Bed bugs impact tourism and means of transportation, this latter being a factor that contributes to the spread of infestations. Economic implications are also substantial for the residents and the management office of infested buildings. Due to their ability to hide in cracks and crevices and to survive without feeding over several months, bed bugs are hard to control, and often require more than one intervention. Although physical control measures such as heat, steam, vacuuming are important in integrated pest management programs for bed bugs, control of this pest mostly relies on the application of insecticides in their environment. The most widely used insecticides are the pyrethroids and the organophosphates. Nevertheless, insecticide resistance in bed bugs appears to increase dramatically in the USA and other parts of the world (Boase & Naylor, 2006; Moore & Miller, 2006; Romero *et al.*, 2007; Yoon *et al.*, 2008; Kilpinen *et al.*, 2011). Reports of pyrethroids resistance remain rare in Europe (Boase *et al.*, 2006; Kilpinen *et al.*, 2011). The determination of resistance status of bed bugs is usually performed by topical application tests or tarsal exposure tests. V419L and L925I mutations in a voltage-gated sodium channel a-subunit gene of *Cimex lectularius*, have also been used as molecular marker of resistance to pyrethroids in a few studies (Yoon *et al.*, 2008; Seong *et al.*, 2010; Zhu *et al.*, 2010).

In France, there is limited information on the status of resistance of bed bugs to pyrethroids. Private pest control practitioners and municipalities report soaring number of calls from individuals or institutions. We evaluated the infestation by bed bugs in a complex of two buildings in the suburb of Paris and assessed their susceptibility to pyrethroid insecticides, using mortality bioassay and molecular methods. The study aimed to contribute to the management of the infestation in these buildings.

## Materials and Methods

### Study Site

The study took place from February to June 2011 in Saint-Ouen, a city bordering the north of Paris. Various ethnic communities are represented among the 45,600 inhabitants of this city. The rate of social housing in the city reaches 45 %. The study site was two 16-story apartment buildings, separated by 30 meters, located in the south of Saint-Ouen. The buildings have 198 apartments (102 in building A and 96 in building B), ranging from one to six rooms. At time of the study, the buildings harboured 473 residents (288 in building A and 185 in building B).

The residents of the building complex complained vigorously to the management office due to heavy and long lasting infestations (for several years according to the management office) of their housing by bed bugs. The owner of the place called the Regional Health Agency to find out an answer to the problem. Our laboratory was contacted by the Regional Health Agency to evaluate the level of infestation and the inconvenience for residents, and to test the susceptibility to pyrethroid insecticides that were occasionally used in the site.

### Survey of the Bed Bugs Infestation

A newsletter about the study has been sent to all residents of both towers. Visual inspection by trained staff and resident interviews were performed about the history of the infestation, past control efforts, and resident awareness of bed bugs in their apartments. The duration of inspection of the apartments varied from around 30 min for the smallest to more than two hours for the largest. The beds, sofas, other upholstered furniture, wheelchairs, perimeter of the doors, curtains, and boxes stored under the beds or in the closets were closely inspected. Beds were disassembled if possible for inspection.

### Collection of Insects

All bed bugs (except the egg stage) were handremoved with flexible forceps (to not harm them) during inspections, and placed in 5 mL haemolysis vial together with a piece of folded bound paper, representing an artificial shelter to avoid excessive mortality of bed bugs in the following hours.

### Mortality Bioassay

The number of bed bugs tested varied according to the level of infestation of the apartments. When less than ten insects were collected from an apartment, all bed bugs were submitted to the effects of a pyrethroid insecticide. When more than ten insects were collected from an apartment, the sample was divided in two halves; one half of bed bugs was submitted to the effects of a pyrethroid insecticide and the other half was placed in contact with isotonic sodium chloride solution as a control. A standardized method for *ex vivo* assessment of efficacy of insecticide products was used as previously described (Chosidow *et al.*, 1994). Briefly, Whatman grade n°1 filter papers of 8.5 cm diameter were impregnated with 0.5 mL of a lotion containing a combination of two pyrethroids, Neopynamine and Sumithrin, at a concentration of 2.69 g.L^-1^ for each compound (commercially available as A-PAR®, Omega Pharma, Châtillon, France). Impregnated papers, containing 474 mg of both active ingredients/m2, were placed in Petri dishes of the same diameter and non-injured alive bed bugs were placed onto impregnated paper and trapped within the closed Petri dishes. The impregnated papers were not allowed to dry out before the test. Isotonic sodium chloride solution was used to impregnate Whatman filter paper as control group. Bed bugs were maintained in an incubator at 24 ± 2 °C and 55-65 % relative humidity. Bed bugs were examined for activity under a binocular loupe one hour and 24 h after insecticide or isotonic sodium chloride solution exposure. Bed bugs were judged as «dead» if there were no vital signs or minor vital signs present (merely internal gut movements, movements of antennae, minimal leg movements with or without stimulation by a forceps).

### DNA Extraction

Adult bed bugs were individually processed for DNA extraction according to the following procedure. Each bed bug was cut in four pieces using a sterile scalpel. One of these pieces was placed in a sterile microfuge tube, incubated at 65 °C for 15 min and crushed using Tissue Lyser π(Qiagen, Germany) for 8 min at 30 Hz frequency. The sample was homogenized in 180 μL of buffer ATL and 20 μL of proteinase K (QIAamp DNA Mini Kit, Qiagen, Courtaboeuf, Germany), vortexed for 20 s and then incubated for three hours at 56 °C. Further steps were done according to the manufacturer’s instructions.

### Genotyping of *KDR*-Like Gene

The amplification of 474-bp and 744-bp portions of the voltage sensitive sodium channel-subunit gene (*kdr*-like gene) spanning the codon 419 (fragment 1) and 925 (fragment 2) respectively was performed on 2 μL of DNA solution under the following conditions. In a 25 μL reaction mixture containing 0.3 μmol/L of each primer: 5’AACCTGGATATACATGCCTTCAAGG3’ (forward) and 5_TGATGGAGATTTTGCCACTGATG3_ (reverse) for fragment 1; 5_GGAATTGAAGCTGCCATGAAGTTG3_ (forward) and 5_TGCCTATTCTGTCGAAAGCCTCAG3_ (reverse) for fragment 2, 200 μmol/L dNTPs, buffer (50 mmol/L KCl, 10 mmol/L Tris-HCl, pH 8.3, and 1 mmol/L MgCl2), and 2.5 U of Thermus aquaticus DNA polymerase (AmpliTaq Gold; PerkinElmer Life and Analytical Sciences, Boston, MA). The sample was incubated for 10 min at 95 °C for denaturation before cycles (94 °C × 40 s, 52 °C × 40 s (fragment 1) / 55 °C × 40 (fragment 2), and 72 °C × 40 s). After 40 cycles, primer extension was continued for 10 min at 72 °C. PCR products purification and DNA sequencing were performed by the Qiagen laboratory (Qiagen, Courtaboeuf, Germany). Electrophoregrams were visualized and analyzed with the Auto assembler program (Applied Biosystem). The obtained sequences were compared with wild-type sequences (GenBank accession numbers, GU123927 and GU123928). The presence of single nucleotide polymorphisms (V419K and L925I) was confirmed by reading both forward and reverse strands.

### Ethics Statement

The agreement and the authorization of the owner of the place were obtained for the study. No consent from the residents was required for this study. The residents of this social housing had a legal obligation to participate in the program of bed bugs eradication.

## Results

### Survey of the Bed Bug Infestations

**Fig. 1.**
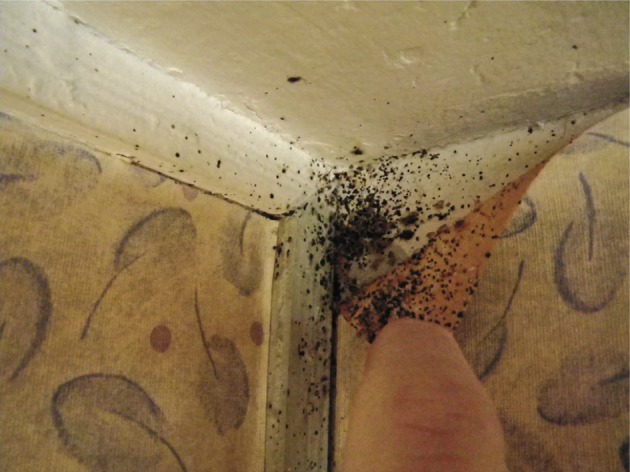
Adult bed bugs and excreta in a flat.

**Fig. 2.**
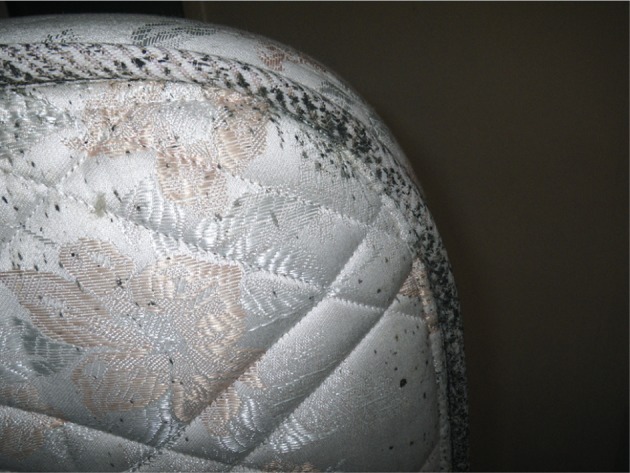
Bed bugs excreta on a mattress.

**Fig. 3.**
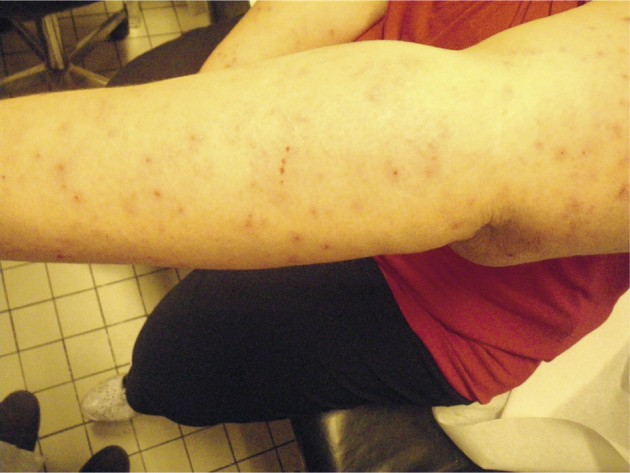
Lesions on a human arm caused by bed bugs bites.

In all, 99 out of 102 apartments have been visited in building A and 93 out of 96 in building B (Table I). In building A, 24 apartments had live bed bugs (Fig. 1). Obvious clues of a current or past presence of bed bugs (excreta of bed bugs on a mattress for example) were found in 35 other apartments in which no bed bug have been found during the inspection (Fig. 2). 90 residents lived in infested apartments. Among them, 49 (54.4 %) reported bed bugs bites (Fig. 3). 38 residents (42.2 %) lived in infested apartments but did not notice bites. Three other subjects (3.4 %) lived alone in an infested apartment and thus were considered as being “insensitive” to bites. In building B, only two apartments had live bed bugs. Obvious clues of a current or past presence of bed bugs were found in 15 other apartments in which no bed bug have been found during the inspection. Only seven residents lived in infested apartments: four (57 %) complained about bed bugs biting and three (43 %) did not notice bites. In summary, the complex of buildings was heavily infested: 39.6 % of apartments (59.6 % of inspected apartments in building A and 18.3 % in building B) harbored bed bugs during inspection or in a recent past.

**Table 1.** Infestation of the two buildings by bed bugs.

	Building A	Building B	Total
Number of apartments	102	96	198
Number of visited apartments (%)	99 (97)	93 (96.9)	192 (97)
Number of residents	288	185	473
Infested apartments (%)	24 (24.2)	2 (2.1)	26 (13.5)
Suspected infested apartments (%)	35 (35.3)	15 (16.1)	50 (26)
Infested or suspected infested apartments (%)	59 (59.6)	17 (18.3)	76 (39.6)
Residents in infested apartments	90	7	97
Residents bitten in infested apartments (%)	49 (54.4)	4 (57)	53 (54.6)
Residents not bitten in infested apartments (%)	38 (42.2)	3 (43)	41 (42.3)
Residents bitten in infested apartments but insensitive (%)	3 (3.4)	0 (0)	3 (3.1)

### Collection of Insects

A total of 548 and 16 bed bugs of all stages (except eggs) were collected in infested units of building A and B, respectively. The number of insects collected varied according to apartments from only one to more than 30. Despite our precautions, 294 bed bugs were dead before their inclusion in insecticide resistance tests, which were performed within 12 hours after collection.

### Mortality Bioassay

Only adult bed bugs were selected for the test. In all, 215 bed bugs were submitted individually to the test. Seventy-two bed bugs were used as controls, of which only one was dead at the end of the test. Among the 143 bed bugs exposed to insecticide, 54 (37.8 %) were reported as resistant (95 % confidence interval: 29.9-45.7 %) and 89 (62.2 %) were susceptible (95 % confidence interval: 54.3-70.1 %). Most resistant bed bugs (37 out 54) were collected in two highly infested apartments. Eight apartments harbored at least one resistant bed bug.

### Genotyping of *KDR*-Like Gene

**Fig. 4.**
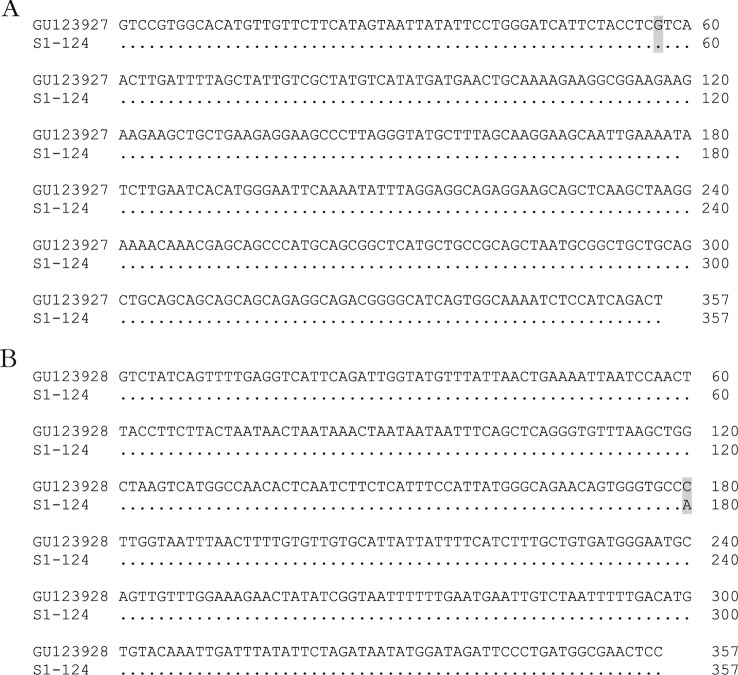
A: alignment of a part of the sequence of the voltage-gated sodium channel a-subunit gene, encompassing codon 419 (highlighted in yellow), from a pyrethroid-susceptible bed bug [Genbank access number: GU123927] and 124 adult bed bugs collected in the study. Nucleotides identical to those of the GU123927 sequence are indicated by periods; B: alignment of a part of the sequence of the voltagegated sodium channel a-subunit gene, encompassing codon 925 (highlighted in yellow), from a pyrethroid-susceptible bed bug [Genbank access number: GU123928] and 124 adult bed bugs collected in the study. Nucleotides identical to those of the GU123928 sequence are indicated by periods.

The DNA of all 143 bed bugs that have been exposed to insecticide during the mortality bioassay was extracted. Both parts of *kdr*-like gene encompassing codons 419 and 925 were successfully amplified and sequenced for 124 bed bugs (Fig. 4), corresponding to 75 susceptible and 49 resistant bed bugs according to the bioassays. All sequenced bed bugs had homozygous wild-type V419 codon and homozygous mutated L925I codon of *kdr*-like gene.

## Discussion

The complex of buildings appeared heavily infested with about 40 % of infested apartments in mean. This prevalence did not necessarily reflect the situation in the whole city of Saint-Ouen or other cities around Paris. This prevalence was comparable to that found (45 %) in a recently published study which was performed in a 15-story tower of Indianapolis, USA, including 223 one-bedroom apartments (Wang *et al.*, 2010). Building A was much more infested than building B, though both buildings were located in a very similar environment. We can hypothesize that building A was infested earlier than building B, which could explain the larger spreading of bed bugs in the first tower. Other factors may also intervene as both towers were not identical in terms of distribution of apartments. Thus, building A harboured a great number of large families with relatively low income in vast apartments. Building B had more small apartments, harboring couples or families of less than five persons. The apartments of building B had also a better aspect (maintenance, cleanness, tidying up). We can only assume the origin of the infestation. It seemed that many residents bought second-hand furniture in the flea market of Saint-Ouen, a globally renowned market including second-hand furniture stores, located at only two hundred meters of the complex building. Those furnitures are suspected to frequently carry over bed bugs in new housings.

During the study, we have inspected 192 apartments out of 198 (97 %) of the complex building and interviewed their residents. There was no empty flat in the complex building but a few residents were repeatedly absent or could not be reached. Others refused obstinately to participate despite a notification of legal obligation. Residents have been interviewed in almost all apartments. Among residents living in infested apartments, more than a half complained about bed bugs bites. Some residents developed more important reactions than others (Fig. 3) but there was no report of severe clinical signs to our knowledge. Bed bugs bite at night when their hosts are sleeping. The usual response to a bed bug bite appears to be no reaction with a barely visible punctum at the location of the bite (Delaunay *et al.*, 2011). Otherwise, cutaneous reactions may vary from none to bullous rashes but the common dermatological presentations are 2- to 5-mm itchy maculopapular, erythematous lesions. Lesions usually resolve spontaneously within two-six weeks. Thus, bed bug bites may have been unnoticed by residents and the number of individuals living in infested apartments and who were considered as not bited was probably overestimated. Conversely, bites of other origin (mosquitoes or fleas for example) could have been misleading in some occasions.

Bioassays showed that pyrethroids resistant bed bugs were present in many apartments. Pest control operators rarely use pyrethroid insecticides in Paris and its suburb. They use mostly carbamate compounds as bendiocarb (Ficam W®, Bayer Environmental Science, Lyon, France). These products are sold only to professional pest control operators. Four applications of bendiocarb were done by pest control operators in the buildings within two years before we collected bed bugs. Those applications were single treatment, separated by six months in mean. Conversely, private individuals frequently use pyrethroid insecticides, which are easily available in stores. As a result, pyrethroid insecticides are often improperly used in terms of concentrations and number of applications, which may induce unnecessary insecticide pressure to bed bugs and other pests.

Contrary to other studies, we have performed bioassays directly on bed bugs collected on the field, and not in adapted colonies fed several weeks or months in the laboratory. Thus, the experiments on insecticide resistance were performed in a quasi real-world setting that approximates what residents and bedbugs would experience. The spontaneous mortality of bed bugs observed within 12 hours after their capture was high (52.1 %). This could be due to unnoticed injuries or stresses done during the capture and/or transportation to our laboratory. Previous aggressions of bed bugs may have also be done before their capture (insecticides or other means of control). This relative fragility of bed bugs may have led to an overestimation of the activity of pyrethroids. Using tarsal exposure test to commercial formulations of permethrin and deltamethrin, Kilpinen *et al.* reported recently high resistance rates in bed bugs populations collected in private homes in Denmark and fed on a human volunteer 1-2 weeks before testing (Kilpinen *et al.*, 2011). These data and ours showed that resistance to pyrethroids is present in at least two distant countries in Europe. As pyrethroids are among the most widely used insecticides by people against bed bugs in France and other countries, these results are worrying.

An identical *kdr*-like gene haplotype, homozygous mutated L925I codon and homozygous wild-type V419 codon, was found in all bed bugs. This haplotype corresponded to bed bugs reported as resistant according to previous studies (Yoon *et al.*, 2008; Seong *et al.*, 2010; Zhu *et al.*, 2010). To our knowledge, our data are the only results of genotyping of *Cimex lectularius kdr*-like gene in Europe. This haplotype was already found by Zhu *et al.* in bed bug populations collected in 2006 and 2007 in various cities of USA (haplotype B) (Zhu *et al.*, 2010). Using bioassays, those authors evaluated the susceptibility to deltamethrin of three bed bugs populations having this haplotype, which revealed a resistant status. Our data showed less clear-cut association with bioassays results with 38 % of resistant bed bugs. Contrary to previous studies, we exposed bed bugs to an association of two pyrethroids, which may have enhanced the apparent insecticide activity. In addition, the proportion of susceptible bed bugs may have been overestimated due to the poor general condition of some of the collected insects, though bed bugs exposed to isotonic sodium chloride solution as control groups did not reveal such spontaneous mortality during the test. The V419L mutation could play a role in pyrethroid resistance, but less than the L925I mutation which has been selected more intensively than the V419L mutation and which probably plays a central role in resistance to pyrethroids (Seong *et al.*, 2010). Resistance to pyrethroids was not always associated with the V419L mutation in populations from the USA and this mutation was not always associated with the L925I mutation to form a resistant haplotype (Yoon *et al.*, 2008; Seong *et al.*, 2010). The bed bugs tested in our series presented a genomic resistance to pyrethroids by possessing the L925I mutation at a fixation rate. Further studies are required to know whether bed bugs having both mutations are more resistant or not than those having only the L925I mutation. Nevertheless, our results advocated to not recommend the use of pyrethroids as insecticide for the control and eradication of bed bugs in this site. Results of the present study showed the presence of pyrethroids resistance in the site and warrant to monitor the susceptibility to insecticides of bed bugs in the future.
